# Prevalence of problematic smartphone usage and associated mental health outcomes amongst children and young people: a systematic review, meta-analysis and GRADE of the evidence

**DOI:** 10.1186/s12888-019-2350-x

**Published:** 2019-11-29

**Authors:** Samantha Sohn, Phillipa Rees, Bethany Wildridge, Nicola J. Kalk, Ben Carter

**Affiliations:** 1 Institute of Psychiatry Psychology and Neuroscience, King’s College London, London, UK; 2 Institute of Child Health, University College London, London, UK; 3 Department of Addictions, Institute of Psychiatry Psychology and Neuroscience, King’s College London, London, UK; 4 South London and Maudsley NHS Foundation Trust, London, UK; 5 Department of Biostatistics, and Health Informatics, Institute of Psychiatry, Psychology and Neuroscience, King’s College London, Denmark Hill, De Crespigny Park, London, SE5 8AF UK; 6 Cochrane Skin Group, School of Medicine, Nottingham University, Nottingham, Nottinghamshire, UK

**Keywords:** Problematic smartphone usage, Anxiety, Depression, Sleep, Educational attainment

## Abstract

**Background:**

Over the past decade, smartphone use has become widespread amongst today’s children and young people (CYP) which parallels increases in poor mental health in this group. Simultaneously, media concern abounds about the existence of ‘smartphone addiction’ or problematic smartphone use. There has been much recent research concerning the prevalence of problematic smartphone use is in children and young people who use smartphones, and how this syndrome relates to mental health outcomes, but this has not been synthesized and critically evaluated.

**Aims:**

To conduct a systematic review and meta-analysis to examine the prevalence of PSU and quantify the association with mental health harms.

**Methods:**

A search strategy using Medical Subject Headings was developed and adapted for eight databases between January 1, 1st 2011 to October 15th 2017. No language restriction was applied. Of 924 studies identified, 41 were included in this review, three of which were cohort studies and 38 were cross sectional studies. The mental health outcomes were self-reported: depression; anxiety; stress; poor sleep quality; and decreased educational attainment, which were synthesized according to an a priori protocol.

**Results:**

The studies included 41,871 CYP, and 55% were female. The median prevalence of PSU amongst CYP was 23.3% (14.0–31.2%). PSU was associated with an increased odds of depression (OR = 3.17;95%CI 2.30–4.37;*I*^2^ = 78%); increased anxiety (OR = 3.05 95%CI 2.64–3.53;*I*^2^ = 0%); higher perceived stress (OR = 1.86;95%CI 1.24–2.77;*I*^2^ = 65%); and poorer sleep quality (OR = 2.60; 95%CI; 1.39–4.85, *I*^2^ = 78%).

**Conclusions:**

PSU was reported in approximately one in every four CYP and accompanied by an increased odds of poorer mental health. PSU is an evolving public health concern that requires greater study to determine the boundary between helpful and harmful technology use. Policy guidance is needed to outline harm reduction strategies.

## Background

Over the past decade there has been an increase in use of smartphones among children and young people (CYP) [1, 2] which has occurred at the same time as a rise in common mental disorders in the same age group, including reported depressive symptoms, poor sleep and suicide ideation [3–5] with grave implications for life-long mental health [6, 7] and the healthcare economy [8].

Smartphones became widely available in 2011, since then usage has increased. Smartphone ownership in children aged 11 and older is ubiquitous, and the prevalence of mental health problems peaks during the teenager years [2]. There is a public health uncertainty regarding a possible association between smartphone use and mental health in CYP, and in the UK, policy making has been hindered by a paucity of evidence. Explicitly the debate in the literature has concerned the relationship between amount of screen time, or amount of smartphone use, in CYP and clinically defined, mental health outcomes, with some studies reporting no association and others exhibiting a clear association [9, 10]. One challenge is the date when the studies were carried out, often before the advent of widespread smartphone use, meaning the term screen-time may include televisions or personal computers, although it has a more common interpretation as a smartphone today [11]. Other limitations include that longer use is assumed as harmful, and this may not necessarily be accurate.

One possibility of the conflicted findings may be that it is not smartphone use per se that is associated with poor mental health, but particular patterns of smartphone-related behaviour. Both the mainstream media and researchers have raised the possibility that people can become addicted to smartphone use, though in the academic realm, this is controversial [12]. Nonetheless, recent years have seen an explosion in research considering the prevalence of problematic smartphone use (PSU), which has been operationalised in such a way that it maps onto concepts of behavioural addiction: tolerance, withdrawal (dysphoria when the battery dies), preoccupation, neglect of other activities, subjective loss of control and continued use despite evidence of harm [13–18]. Other behavioural addictions, such as problem gambling, show robust associations with common mental disorders such as depression [19],where sporadic gambling does not. If a distinctive problematic pattern of smartphone use can be demonstrated to be prevalent, and if this pattern of use is associated with harm, there is value in identifying children and young people with this pattern of use and potentially addressing it clinically. Given the large increase in research studies using tools to estimate the prevalence of PSU (and examine mental health associations), it is now appropriate to evaluate the evidence.

### Objectives

Despite concerns about the impact of smartphones on the mental health and wellbeing of CYP, we are unsure of the prevalence of PSU amongst this cohort, and causal associations between PSU and poor mental health have yet to be established. We therefore undertook a systematic review and GRADE of the evidence with the primary aim of characterising the prevalence of PSU amongst CYP, with smartphones as the exposure, and PSU as the outcome. We also undertook a meta-analysis with the secondary objectives of: assessing sociodemographic characteristics associated with PSU; quantifying the impact of PSU on: mental health outcomes; sleep; and school performance. Mental health outcomes assessed included any reported measure of depression or anxiety (diagnosis or screening questionnaire), and perceived stress; sleep quality. In addition, school performance was included as a measure of functional impairment in this population.

## Methods

### Study selection

The systematic review was carried out according to the PRISMA statement and reported with the PRISMA checklist [20]; furthermore an a priori protocol is registered on PROSPERO (#88800). We included randomised controlled trials; cohort; cross-sectional; and case-control studies. Eligibility criteria included studies of mobile device exposure focusing on children and young people (with a mean population age of no greater than 25) [21]. This broader definition of CYP (recently proposed by Sawyer et al.) was specifically chosen, as it is more inclusive of the CYP population who are developmentally vulnerable to problems such as PSU, and also so as to not overlook important data relevant to the paediatric population. Included studies needed to use a scale with a clear threshold to define PSU. Studies that investigated particular uses of smartphones, such as gambling or gaming, were excluded, as these activities have been identified as addictive in and of themselves [22].

### Data sources and search strategy

Searches were carried out from January 1st 2011 to October 15th 2017, with no language restriction. This time restriction was specifically chosen to capture studies of current and modern smartphone technology [23]. A search strategy based on the MeSH headings ‘cell phone’, ‘behaviour, addictive’, and ‘adolescent’ (See Additional file 1: Table S1), was applied to 8 databases, including Scopus, Web of Science, ScienceDirect, PubMed, Medline, CINAHL, PsychInfo, and EMBASE, on October 17th, 2017. Two independent researchers (SS, BW) screened the results from the search strategy, and the full texts of all studies that meet these criteria were then further assessed for eligibility. Any disagreements were resolved by discussion with a senior researcher (BC). Additional studies were identified by reviewing the reference sections of relevant papers.

### Quality assessment and characteristics of included studies

Studies were assessed for methodological quality using a modified Newcastle-Ottawa scale separately for each study design, where each study was assessed and deemed as high, unclear, or low risk of bias across three domains (selection, comparability and outcomes) [24] (See Additional file 1: Table S2). The quality of evidence across the included studies was assessed using GRADE methodology [25]. Study characteristics extracted included: year of study; geographical region; instruments used; response rate; reported prevalence of PSU; mental health; and educational attainment. Study authors were contacted in cases of incomplete data.

### Problematic smartphone usage (PSU)

We defined PSU in accordance with the literature as smart phone use associated with at least some element of dysfunctional use, such as anxiety when the phone was not available, or neglect of other activities [13, 18]. This was measured by included studies using a range of scales, such as the Smartphone Addiction Scale (SAS) or the Mobile Phone Problematic Use Scale (MPPUS) [13, 14]. We summarise each of the instrument definitions used, and highlight the behavioural domains in Additional file 1:Table S4 and S5.

### Data synthesis

#### Estimating the prevalence of PSU

The primary objective was to estimate the prevalence of PSU amongst CYP. The validated thresholds developed by each of the the scales were applied, and this was summarised with a median and interquartile range.

#### Association between the prevalence of PSU and common mental health outcomes

The secondary objectives were to investigate PSU associated with the following outcomes: depressed mood; anxiety; stress; poor sleep quality; and educational attainment. A summary of the PSU findings from the studies were assessed using: logistic regression odds ratio (ß); correlation (r); or a Chi-square test.

Where study design, level of exposure of PSU, and outcomes were homogeneous, outcome data were included in a pooled random-effects meta-analysis using the Mantel-Haenzsel method [26, 27]. Where studies reported logistic regression analyses, the analysis data were pooled with dichotomous data using the generic inverse variance method. Pooled odds ratios (OR) are presented with 95% confidence intervals (95%CI), *p*-values, and *I*^2^ heterogeneity statistics. Revman 5.3 was used to conduct the analysis.

### Assessment of subgroups and statistical heterogeneity

Heterogeneity exceeding 85% was explored using subgroup analyses [23]. Pre-determined subgroup analyses included: study quality assessment; age; gender; high PSU prevalence (> 40%); time period of study; and geographical region.

### Changes since the protocol was registered

After protocol registration, the following additional outcomes were included: suicidal ideation, and associated psychological factors.

## Results

### Identified studies and quality assessment

**Fig. 1:**
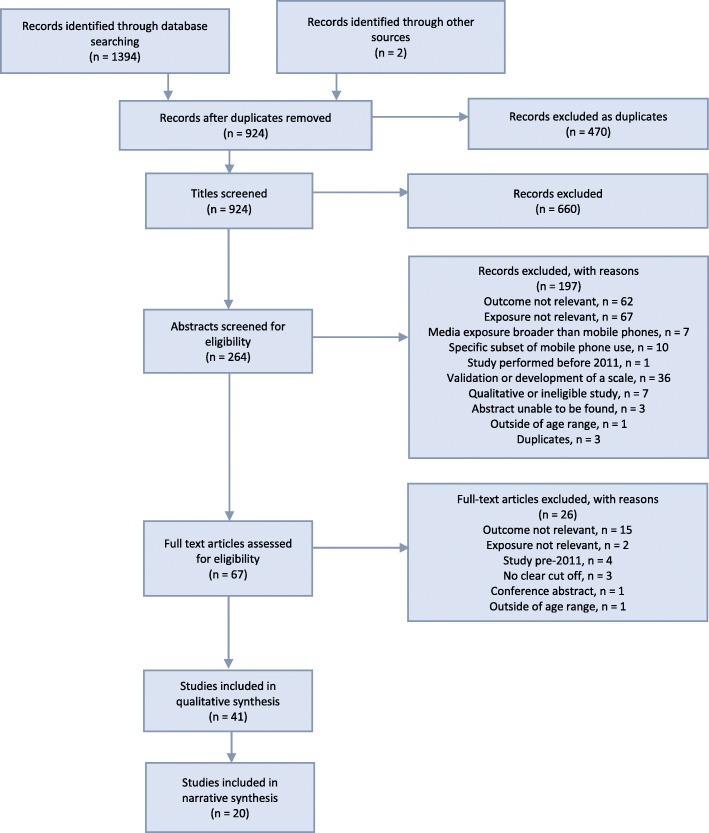
The PRISMA Flow chart

Of 924 studies identified, 41 studies were included in this review (Fig. 1). Of those, 22 studies were deemed to be of poor methodological quality, and 19 of moderate quality (See Additional file 1: Table S2). Three cohort and 38 cross-sectional studies were included, with 41,871 participants, 55% of which were female. Included studies were conducted in Europe (*n* = 9), Asia (*n* = 30), and America (*n* = 2) (See Additional file 1: Table S3). There was wide variability in the definitions of PSU (See Additional file 1: Table S4), and the criteria used ranged from a single criterion such as psychological withdrawal phenomena (*n* = 2), to measurement of tolerance, withdrawal, loss of control, preoccupation, neglect of other activities and evidence of harm, which form the criteria for behavioural addictions (*n* = 19) (See Additional file 1: Table S5).

### Types of PSU usage

Communication was the most frequent type of smartphone usage by those with PSU, reported by 14 studies [28–39]. Problematic users reported that social networking was the most important or preferred activity on smartphones [34, 40]. ‘Addicted’ or ‘problem user’ groups were linked with particular phone uses: TV watching [35] and social networking [37, 38]. Lee and Lee [41] found that use of phones to gain peer acceptance was the most significant type of use related to PSU.

Both substance use and other behavioural addictions were associated with PSU. Internet addiction [33, 42–44], Facebook addiction [31], compulsive buying [43], increased alcohol use [42], and cigarette smoking [42] were also found to be positively associated with PSU.

### Sociodemographic characteristics associated with PSU

Across 14 studies, age was correlated with PSU [28–33, 40–42, 45–49], and 17 to 19 year-olds were the most frequent sufferers of PSU. Females were reported as more prone to PSU by 13 studies [31–34, 40, 41, 48–54]; however 4 studies reported the opposite [35, 46, 55, 56]. PSU in males was correlated with use of media applications and games, while in females it was correlated with communication and social networking applications [28]. PSU was also positively associated with monthly cost of living [28], family income [36], and a higher economic status [42].

### Estimating the prevalence of PSU

Prevalence was assessed using 24 different questionnaires, with the most common being the Smartphone Addiction Scale, Short Version (SAS-SV; *n* = 7) and the Smartphone Addiction Proneness Scale (SAPS; *n* = 5), for further details (See Additional file 1: Table S4).

**Fig. 2:**
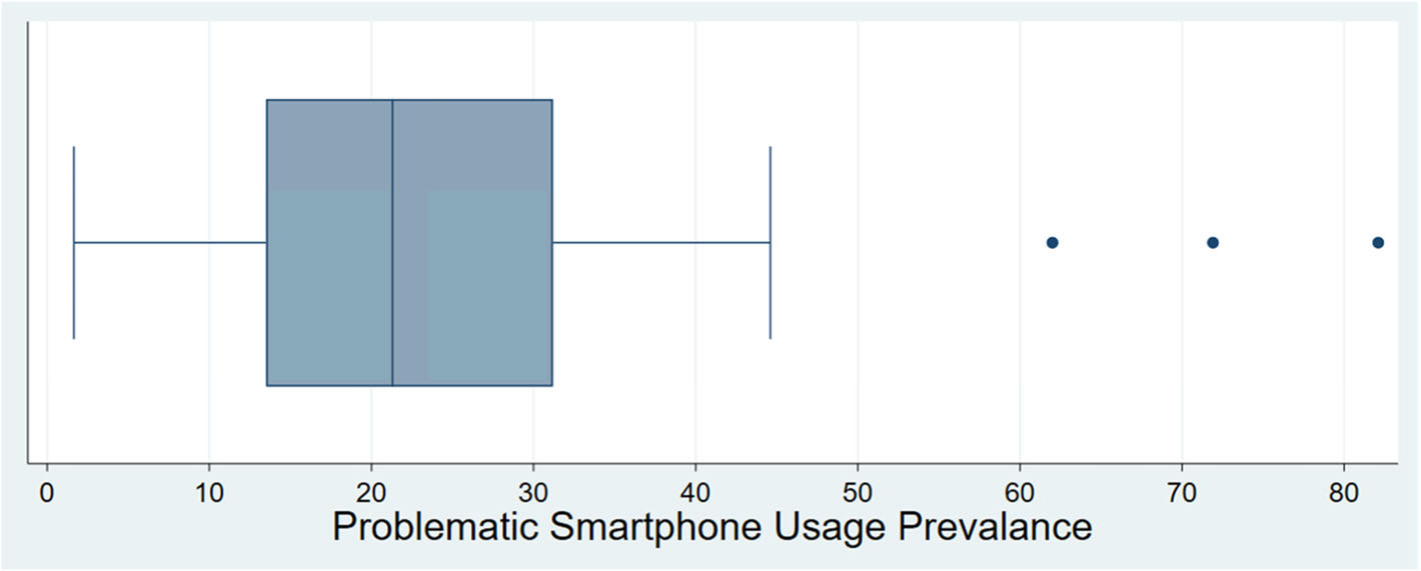
A boxplot of Problematic Smartphone Usage (PSU) Prevalence

The majority of studies (*n* = 31) found a prevalence between 10 and 30%, and the median was 23.3% (interquartile range 14–31%, Fig. 2).

### PSU associated with mental health outcomes

PSU has been consistently associated with measures of poor mental health, in particular relating to depression, anxiety, stress, poor sleep quality, and day to day functional impairment demonstrated by poor educational attainment. Of the studies included, 20 investigated the relationship between PSU and mental health amongst CYP. This is summarized in a qualitative synthesis (See Additional file 1: Table S6).

#### Depression

**Fig. 3:**
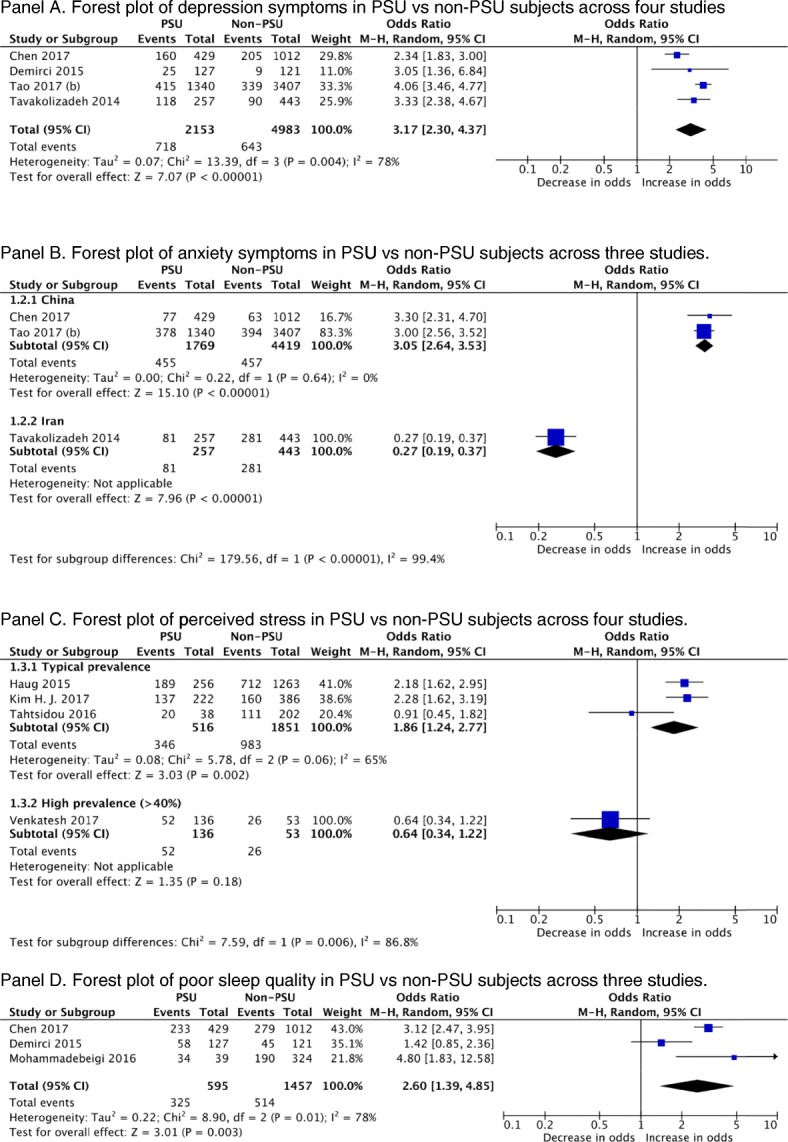
A meta-analysis of Problematic Smartphone Usage (PSU) and mental health outcomes of: Depression (Panel A); Anxiety (Panel B); Percieved Stress (Panel C); and Poor Sleep (Panel D)

Eight studies [28, 36, 48, 57–61] reported a significant association between PSU and depression across 10,099 participants. Dichotomous data from four studies was extracted using standard cut-offs for the clinical diagnosis of depression. In those with PSU the odds ratio (OR) of depression was 3.17 (95% CI, 2.30, to 4.37; *p* < 0.001; *I*^2^ = 78%; Fig. 3). Given the consistency of the study findings, we have upgraded this to a GRADE of moderate quality.

#### Anxiety

Seven studies [28, 30, 36, 57, 59–61] investigated the relationship between PSU and anxiety in CYP. Of seven studies across 9359 participants, six found a significant positive association between PSU and anxiety; one study reported a negative association [60]. The pooled OR for anxiety amongst CYP with PSU was 2.60 (95% CI 1.39, to 4.85; *p* < 0.001; *I*^2^ = 78%). The large heterogeneity due to Tavakolizadeh et al. [60], is explained by geography, and the Iranian protests of 2011–2012. After accounting for the heterogeneity, the OR for anxiety amongst CYP with PSU was 3.05 (95% CI 2.64, to 3.53; *p* < 0.001; *I*^2^ = 0%;. 3) The GRADE of evidence was categorised as low quality.

#### Stress

Five studies [36, 39, 40, 44, 62] investigated perceived stress across 3618 participants. Four studies found a significant association between PSU and perceived stress amongst CYP, whilst Tahtsidou et al. [44] found no significant relationship. A subgroup analysis was introduced due to PSU prevalence. Most heterogeneity was accounted for by Venkatesh et al. [39], a study reporting a PSU prevalence of 71.9% – this study was subsequently excluded. The pooled OR for typical PSU prevalence and perceived stress amongst CYP was 1.86 (95%CI 1.24, to 2.77; *p* = 0.002; *I*^2^ = 65%; Fig. 3). The GRADE of the evidence was categorised as low quality.

#### Sleep

There were seven studies [28, 35, 57, 61, 63–65] which investigated poor sleep across 4194 CYP. Six studies reported a significant positive association between PSU and poor sleep, while Demirci et al. [58] reported no significant association. The pooled OR for the extracted data on PSU and subsequent poor sleep was 2.60 (95%CI, 1.39, to 4.85, *p* = 0.003, *I*^2^ = 78%; Fig. 3). The GRADE of evidence was categorised as low quality after accounting for both the narrative analysis and the pooled analysis.

#### Educational attainment

Six studies [41, 48, 50, 60, 66, 67] explored PSU and educational attainment across 6655 CYP. Four studies reported a significant association between PSU and poor educational attainment, whilst one [60] found no significant relationship.

Variations in measures of educational attainment were used; it was therefore not appropriate to pool the results of the studies. However, they are summarised to demonstrate the consistency of reported associations between PSU and poor educational attainment (See Additional file 2: Figure S1).

#### Suicide

One study reported an increased odds of suicidal ideation amongst those with PSU [62]; however, this was assessed through a single screening question and caution should be taken with this finding.

#### Psychological factors associated with PSU

A range of different personality and emotional factors were investigated in relation to PSU. Somewhat paradoxically, traits associated with greater risk-taking (such as low self control, impulsivity, emotional instability, and openness) and traits associated with avoidance of risk taking (such as perfectionism and conscientiousness), were more common amongst problematic smartphone users [51, 52, 61, 66]. An insecure attachment style, loneliness [45, 56, 65], and low self esteem [49] were all associated with PSU.

## Discussion

This is the first systematic review, meta-analysis and GRADE to investigate the prevalence of PSU amongst CYP. The prevalence of PSU amongst CYP was found to be between 10 and 30%, indicating that it is a widespread problem. Females in the 17 to 19-year-old age group were most likely to exhibit PSU. Furthermore, PSU was consistently associated with depression, self-reported anxiety, maintenance insomnia, increased perceived stress, and poor educational attainment. Overall, those with PSU had an increased risk of poor mental health, wellbeing and day-to-day functioning.

### Context of current literature

PSU shares many traits with substance abuse disorders and behavioural addictions [13–18], and it appears to be common. This is unsurprising considering that those at risk of PSU have similar traits to those at risk of other addictions. Like alcohol, smartphone use is socially acceptable and widely available. In addition, smartphones are seen to facilitate work and education, as well as leisure. PSU therefore poses a different and arguably much bigger public health problem than substances of abuse or even Internet gaming. The pathogenesis of PSU is poorly understood and likely complex [45, 68, 69]. Some have suggested that the continued interconnectedness and anticipation of response plays a role [23].

The incidence of mental health conditions amongst CYP has increased substantially over the last ten years, representing a significant burden on healthcare systems worldwide [6, 8, 70, 71]. The reason for this increase in incidence is unknown, but has been most notable amongst adolescent females, the same cohort shown to be most at risk of PSU in our review [5]. This has parallels between the 68% increase in self harm rates in the UK since 2011, at the same time as the widespread introduction of smartphones [72]. Studies have previously suggested that PSU may at least partly underlie this epidemiological shift. Given the frequency of PSU amongst CYP and its significant association with symptoms of common mental disorders, as highlighted by our review, this relationship and consideration of PSU as a potential causative factor requires urgent further exploration.

### Strengths and limitations

This work is strengthened by the inclusion of studies from wide geographical regions that reported consistent and plausible findings. However, given the nature of the review question, studies were non-randomised and at a high risk of bias. Weaknesses of implementation include varying definitions and thresholds for PSU, some of which were incompletely described. Mental health outcomes were all responses to self-report questionnaires rather than formal diagnoses, suicidal acts or referral to secondary child and adolescent mental health service care, raising the possibility that these are sub-threshold symptoms. Furthermore, reverse causality cannot be excluded as rationale for the associations found.

### Implications for policy, practice and research

Our review indicates that approximately 1 in 4 CYP are demonstrating problematic smartphone use, a pattern of behaviour that mirrors that of a behavioural addiction. A consistent relationship has been demonstrated between PSU and deleterious mental health symptoms including: depression; anxiety; high levels of perceived stress; and poor sleep. Younger populations are more vulnerable to psychopathological developments, and harmful behaviours and mental health conditions established in childhood can shape the subsequent life course. Further work is urgently needed to develop assessment tools for PSU, and prevent possible long-term widespread harmful impact on this and future generations’ mental health and wellbeing. In particular, longitudinal studies are required to characterize the causality of the relationships found in this study between PSU and mental health. Possible research could include cohort studies looking at changes in experience of psychopathological symptoms in relation to changes in PSU levels, or a randomized controlled trial comparing the impact of smartphone use, for example in terms of duration or time of day, on mental health outcomes. Future studies should assess the impact of PSU on more objectively evaluated health outcomes, such as depression or anxiety disorders as detected by structured diagnostic instruments (eg the DSM-5 criteria), referrals to secondary mental health services, or primary care psychological therapies services, or prescriptions for medications such as antidepressants.

The prevalence of PSU amongst CYP and its association with symptoms of common mental disorders is a growing public health problem and as such, it should be a concern to policy makers. To address PSU amongst CYP, an accepted and validated diagnostic definition is firstly required, to systematically identify those suffering. Healthcare providers should recognise that excessive or night-time use of smartphones may play a role in the aetiology of mental health and wellbeing problems amongst CYP presenting to their practice. Primary prevention of PSU is difficult given that smartphone use is now a societal norm; however, awareness of the risks of PSU amongst CYP, parents, teachers and healthcare providers could help limit exposure. Further research should develop a consensus regarding the most appropriate diagnostic criteria for PSU, and determine risk factors for PSU. Finally, further exploration of the relationship between PSU and diagnosed mental health conditions is urgently needed to clarify the magnitude of any casual contribution of PSU to the growing burden of mental health conditions amongst CYP.

## Conclusions

Our review indicates that approximately 1 in 4 CYP are demonstrating problematic smartphone use, a pattern of behaviour that mirrors that of a behavioural addiction. A consistent relationship has been demonstrated between PSU and deleterious mental health symptoms including: depression; anxiety; high levels of perceived stress; and poor sleep. Younger populations are more vulnerable to psychopathological developments, and harmful behaviours and mental health conditions established in childhood can shape the subsequent life course. Further work is urgently needed to develop assessment tools for PSU, and prevent possible long-term widespread harmful impact on this and future generations’ mental health and wellbeing.

## Supplementary information

**Additional file 1: Table S1.** Main Search Strategy (from 01/01/2011 to 17/10/2017). **Table S2.** Characteristics of included studies. **Table S3.** Quality assessment of included studies using the Newcastle-Ottawa Scale. **Table S4.** Definitions and Problematic Smartphone Usage terms used, by included studies. **Table S5.** Mapping the instruments used to assess Problematic Smartphone Usage (PSU) onto criteria for behavioural addiction. **Table S6.** Summary of the results of the included studies.

**Additional file 2 Figure S1.** Meta-analyses of Problematic Smartphone Usage (PSU) and the secondary educational outcomes.

## Data Availability

This is an evidence synthesis study, all data is available from the primary research studies, or can be circulated from the corresponding author.

## Abbreviations

CYP: Children and Young People
PSU: Problematic Smartphone Use
