# Correction to: Prevalence of problematic smartphone usage and associated mental health outcomes amongst children and young people: a systematic review, meta-analysis and GRADE of the evidence

**DOI:** 10.1186/s12888-019-2393-z

**Published:** 2019-12-16

**Authors:** Samantha Sohn, Philippa Rees, Bethany Wildridge, Nicola J. Kalk, Ben Carter

**Affiliations:** 1 Institute of Psychiatry Psychology and Neuroscience, King’s College London, London, UK; 2 Institute of Child Health, University College London, London, UK; 3 Department of Addictions, Institute of Psychiatry Psychology and Neuroscience, King’s College London, London, UK; 4 South London and Maudsley NHS Foundation Trust, London, UK; 5 Department of Biostatistics, and Health Informatics, Institute of Psychiatry, Psychology and Neuroscience, King’s College London, Denmark Hill, De Crespigny Park, London, SE5 8AF UK; 6 Cochrane Skin Group, School of Medicine, Nottingham University, Nottingham, Nottinghamshire, UK

**Correction to: BMC Psychiatry (2019) 19:356**

**https://doi.org/10.1186/s12888-019-2350-x**

After publication of our article [1] we were notified that one of the author names was misspelled.

Incorrect author name:

- Phillipa

Correct author name:

- Philippa

The original article has been corrected.

## References

[1] Sohn (2019). Prevalence of problematic smartphone usage and associated mental health outcomes amongst children and young people: a systematic review, meta-analysis and GRADE of the evidence. BMC Psychiatry.

