# Correction to: Prevalence of problematic smartphone usage and associated mental health outcomes amongst children and young people: a systematic review, meta-analysis and GRADE of the evidence

**DOI:** 10.1186/s12888-020-02986-2

**Published:** 2021-01-22

**Authors:** Sei Yon Sohn, Philippa Rees, Bethany Wildridge, Nicola J. Kalk, Ben Carter

**Affiliations:** 1 Institute of Psychiatry Psychology and Neuroscience, King’s College London, London, UK; 2 Institute of Child Health, University College London, London, UK; 3 Department of Addictions, Institute of Psychiatry Psychology and Neuroscience, King’s College London, London, UK; 4 South London and Maudsley NHS Foundation Trust, London, UK; 5 Department of Biostatistics, and Health Informatics, Institute of Psychiatry, Psychology and Neuroscience, King’s College London, Denmark Hill, De Crespigny Park, London, SE5 8AF UK; 6 Cochrane Skin Group, School of Medicine, Nottingham University, Nottingham, Nottinghamshire, UK

**Correction to: BMC Psychiatry 19, 356 (2019)**

**https://doi.org/10.1186/s12888-019-2350-x**

Following publication of the original article [1], the authors identified an error in the author name of Sei Yon Sohn. The previous amendment can be found on 10.1186/s12888-019-2393-z.

The incorrect author name is: Samantha Sohn.

The correct author name is: Sei Yon Sohn.

The author group has been updated above and the original article [1] has been corrected.
