# Supplementary material for: Prevalence of problematic smartphone usage and associated mental health outcomes amongst children and young people: a systematic review, meta-analysis and GRADE of the evidence
Source: BMC Psychiatry. 2019 Nov 29;19:356. doi: 10.1186/s12888-019-2350-x (PMC6883663; doi:10.1186/s12888-019-2350-x)
Supplement: Supplementary file 1 — Additional file 1: Table S1. Main Search Strategy (from 01/01/2011 to 17/10/2017). Table S2. Characteristics of included studies. Table S3. Quality assessment of included studies using the Newcastle-Ottawa Scale. Table S4. Definitions and Problematic Smartphone Usage terms used, by included studies. Table S5. Mapping the instruments used to assess Problematic Smartphone Usage (PSU) onto criteria for behavioural addiction. Table S6. Summary of the results of the included studies. [file 12888_2019_2350_MOESM1_ESM.docx]

Table S1. Main Search Strategy (from 01/01/2011 to 17/10/2017)

| 1 (cell phone OR mobile phone OR telephone, cellular OR portable cellular phone OR smartphone OR portable media device OR tablet phone) (Title, abstract, keywords) |
| --- |
| 2 (dependence OR dependence disorder OR dysfunctional use OR abuse OR over-use OR mis-use OR addiction OR behavior, addictive OR problematic use) (Title, abstract, keywords) |
| 3 (adolescent OR child OR youth OR young adult OR teenagers OR teens OR students OR young person OR juvenile) (Title, abstract, keywords) |
| 4 (1 AND 2 AND 3) |

Table S2. Characteristics of included studies.

| **Source** | **Year of study** | **Study Design** | **Country** | **Questionnaires Sent Out**  **Retrieved**  **Included** | **Response rate** | **Age range (Mean Age +/- SD)** | **% Female** | **Exposure** | **Outcome** | **Prevalence** | **Assessment tool** |
| --- | --- | --- | --- | --- | --- | --- | --- | --- | --- | --- | --- |
| **Bhatt et al., 2017** ^64^ | 2016 | Cross Sectional | India | NA  NA  100 | NA | 20 - 21 (46% of sample) | 54% | Mobile phone | Dependence | 21% | Independent questionnaire (Mobile phone dependence scale) |
| **Bian & Leung, 2015** ^28^ | 2012 | Cross Sectional | China | NA  565  415 | NA | 23 - 26 (60.1% of sample) | 61.60% | Smartphones | Addiction | 13.50% | Independent questionnaire (Smartphone addiction index) |
| **Cerutti et al., 2016** ^73^ | 2013-2014 | Cross Sectional | Italy | 1004  NA  841 | NA | 10 to 16 (12.25 +/- 1.03) | 49% | Mobile phone | Abuse | 26% | Shorter Promis Questionnaire for mobile phone addiction ^74^ |
| **Chen et al., 2017**^29^ | 2016 | Cross Sectional | China | 1556  1556  1441 | 100.00% | 17-26 (19.72 +/- 1.43) | 51.70% | Smartphone | Addiction | 29.80% | SAS-SV* |
| **Chen et al., 2016** ^53^ | 2014 | Cross Sectional | China | 1124  1087  1087 | 96.71% | 20.12 +/- 1.29 | 56.10% | Mobile phone | Addiction | 4.05% | MPAS^£^ |
| **De Sola, 2017** ^30^ | 2014 | Cross Sectional | Spain | NA  1600  1126 | NA | 16 - 65; subset of 16 - 25 (40.9% of sample) | 53.30% | Cell phone | Problematic use | 20.50% | MPPUS^&^ |
| **Demirci et al., 2015** ^48^ | Not reported | Cross Sectional | Turkey | 400  348  319 | 87% | 20.5 ± 2.45 | 63.64% | Smartphone | Addiction | 39.80% | SAS* |
| **Domple et al., 2017** ^31^ | 2016 | Cross Sectional | India | 348  NA  251 | NA | 17-24(19.88+/-1.47) | 47% | Mobile phone | Dependence | 82.10% | Test of Mobile Phone Dependence, Brief ^84^ |
| **Evyazlou et al., 2016** ^61^ | 2014 | Cross Sectional | Iran | 470  NA  450 | NA | 20.4 ± 1.6 | 64% | Mobile phone | Over use | 4.60% | Cell phone overuse scale ^75^ |
| **Garcia-Oliva et al., 2017** ^41^ | 2013 - 14 | Cross Sectional | Spain | NA  NA  319 | NA | 12 to 18 | 48.30% | ICTs/mobile phone | Problematic use | 9.06% | Cuestionario de Experiencias Relacionadas con el Móvil ^76^ |
| **Haug et al., 2015** ^32^ | 2015 | Cross Sectional | Switzerland | 1671  1601  1519 | 95.80% | 18.2 +/- 3.6 | 51.80% | Smartphone | Addiction | 16.90% | SAS-SV* |
| **Hawi et al., 2016** ^42^ | 2014 | Cross Sectional | Lebanon | 339  293  249 | 86.4%% | 17 - 26 (20.96 +/- 1.93) | 45.80% | Smartphone | Addiction risk | 44.60% | SAS-SV* |
| **Hussain et al., 2017** ^33^ | Not reported | Cross Sectional | Global (majority UK, 86%) | NA  871  640 | NA | 13 - 69 (24.89 +/- 8.54) | 65.60% | Smartphone | Problematic use | 2.70% | Independent questionnaire (Problematic smartphone use scale) |
| **Jiang & Shi, 2016** ^57^ | 2015 - 2016 | Cross Sectional | China | 630  NA  601 | NA | 18 - 24 (29.63+/-1.52) | 53.09% | Mobile phone | Problematic use | 8.99% | PMPUS^£^ |
| **Jo, Na, & Kim, 2017** ^34^ | 2016 | Cross sectional | Korea | NA  7200  7003 | NA | 19 - 39 (24.89+/-7.27 M; 23.52 +/-6.13 F) | 55.50% | Smartphone | Addiction proneness | 19.60% | SAPS^∂^ |
| **Kim et al., 2017** ^62^ | 2016 | Cross Sectional | Korea | NA  608  608 | NA | 22.8 | 69.90% | Smartphone | Overuse | 36.00% | SAPS^∂^ |
| **Kwon & Paek, 2016** ^35^ | 2015 | Cross Sectional | Korea | NA  NA  293 | NA | 19 - 29 (20.3 +/- 2.04) | 43% | Smartphone | Addiction Risk | 14.70% | SAPS^∂^ |
| **Lee & Lee, 2017** ^36^ | 2013 | Cross Sectional | Korea | NA  NA  3000 | NA | 14 - 18 | 47.30% | Smartphone | Addiction Proneness | 35.20% | SAPS^∂^ |
| **Lee E. B., 2015** ^37^ | Not reported | Cross Sectional | USA | NA  304  300 | NA | 17 - 55 (22.45 +/- 6.1) | 56% | Smartphone | Addiction | 11% | SAS-SV* |
| **Lee H et al., 2017** ^54^ | 2015 | Longitudinal | Asia | 98  51  35 | 35.71% | 22.3 | 31.43% | Smartphone | Addiction | 28.50% | SAS-SV* |
| **Lee H et al., 2017 (b)** ^43^ | 2014 | Cross Sectional | Korea | NA  421  370 | NA | 13.15 | 50.80% | Smartphone | Addiction | 13.50% | SAPS^∂^ |
| **Lee JE et al., 2017** ^64^ | 2011 - 2013 | Longitudinal | Korea | NA  NA  1125 | NA | 15 (in 2011) | 49.20% | Mobile phone | Addiction | 31.20% | Independent questionnaire |
| **Leung, 2017** ^44^ | Not reported | Cross Sectional | Hong Kong | NA  NA  733 | NA | 11 to 17 | 34.70% | Mobile phone | Dependence | 3.41% | MPDQ^$^ |
| **Li & Lu, 2017** ^65^ | Not reported | Cross Sectional | China | NA  400  396 | NA | 12 to 17 | 49.20% | Mobile phone | Addiction | 27.50% | SQAPMPU^¶^ |
| **Long et al., 2016** ^52^ | 2015 | Cross Sectional | China | 1205  1121  1062 | 93% | 17 - 26 (20.65 +/- 1.54) | 53.86% | Smartphone | Problematic Use | 21.30% | Problematic Cell Phone Use Questionnaire ^85^ |
| **Lopez-Fernandez et al., 2017** ^45^ | 2015 | Cross Sectional | Europe | NA  3604  2775 | NA | 18 - 29 (22.53 +/- 2.84) | 72.80% | Mobile phone | Problematic use | 1.66% | Short Problematic Mobile Phone Use Questionnaire ^77^ |
| **Miguel, 2016** ^40^ | Not reported | Cross Sectional | Spain | NA  NA  775 | NA | 20.7 +/- 3.5 | 87.70% | Mobile phone | Problematic use | 21% | MPPUS^&^ |
| **Mohammadbeigi et al., 2016** ^49^ | 2015 | Cross Sectional | Iran | 380  363  363 | 95.50% | 21.8 +/- 3.2 | 69.10% | Cell phone | Overuse | 10.70% | Cell-phone Over-use Scale ^75^ |
| **Nikhita et al., 2015** ^50^ | 2014 | Cross Sectional | India | 450  NA  415 | 92.20% | 13.99 +/- 0.88 | 39.51% | Mobile phone | Dependence | 31.33% | Mobile Phone Dependence Questionnaire ^78^ |
| **Prasad et al., 2017** ^38^ | 2016 | Cross Sectional | India | NA  588  554 |  | 17 - 35 (21.99+/- 2.95) | 52.90% | Mobile phone | Nomophobia | 24.12% | Independent questionnaire |
| **Rajanna et al., 2016** ^66^ | Not reported | Cross Sectional | India | NA  NA  200 | NA | 13 - 17 | 50% | Mobile phone | Addictive use | 15.50% | Information Technology Addiction survey |
| **Tahtsidou et al., 2016** ^58^ | 2013 | Cross Sectional | Greece | NA  NA  240 | NA | 21.5 | 69.60% | Computer and mobile phone | Dependence | 16.60% | MPDQ^$^ |
| **Tao et al., 2017** ^39^ | 2012 | Cross Sectional | China | 2522  2469  2376 | 94.21% | 19.76 +/- 1.29 | 52% | Mobile phone | Problematic use | 27.90% | SQAPMPU^¶^ |
| **Tao et al., 2017 (b)** ^59^ | 2013 | Cross Sectional | China | 4915  4858  4747 | 96.58% | 19.24 | 58.40% | Mobile phone | Problematic use | 28.20% | SQAPMPU^¶^ |
| **Tavakolizadeh et al., 2014** ^60^ | 2011 | Cross Sectional | Iran | 700  700  700 | 100% | 93.9% from age group 18 - 24 | 44.00% | Mobile phone | Excessive use | 36.70% | MPAI ^79^ |
| **Tossell et al., 2015** ^55^ | Not reported | Longitudinal | USA | NA  NA  34 | NA | Not reported | 44.10% | iPhone | Addiction | 62% | Independent questionnaire (Smartphone Addiction Measurement Instrument) |
| **Venkatesh et al., 2017** ^56^ | 2016 | Cross Sectional | Saudi Arabia | 205  189  189 | 92% | 23.28 male, 23.30 female | 46.56% | Smartphone | Addiction | 71.90% | SAS-SV* |
| **Wang et al., 2013** ^67^ | 2011 | Cross Sectional | China | 2311  2213 | 95.76% | 13 - 29 (21.33 +/- 1.72) | 48.10% | Mobile phone | Dependence syndrome | 23.30% | Independent questionnaire |
| **Warzecha & Pawlak, 2017** ^46^ | 2013 - 2014 | Cross Sectional | Poland | NA  NA  470 | NA | 16 - 19 | 61.10% | Mobile Phone | Addiction | 40.85% (3.83% actually addicted) | Mobile Phone Addiction Assessment Questionnaire (a.k.a. KBUTK) ^80^ |
| **Yildirim et al., 2016** ^47^ | Not reported | Cross Sectional | Turkey | 537  NA  484 | NA | 17 - 34 (20.02 +/- 1.65) | 74.60% | Mobile phone | Nomophobia | 42.60% | Nomophobia Questionnaire ^17^ |
| **Yuchang et al., 2017** ^51^ | Not reported | Cross sectional | China | 305  NA  297 | NA | 17 - 24 (20.24 +/- 1.08) | 45.45% | Smartphone | Addiction | 27.92% | SAS-SV* |

* SAS: Smartphone Addiction Scale; SAS-SV: Smartphone Addiction Scale – Short Version ^14,18^

£ MPAS (Mobile Phone Addiction Scale) or PMPUS (Problematic Mobile Phone Use Scale), due to difference in translation from Chinese^81^

& MPPUS: Mobile Phone Problematic Use Scale ^13^

∂ SAPS: Smartphone Addiction Proneness Scale ^15^

$ MPDQ: Mobile Phone Dependence Questionnaire ^82^

¶ SQAPMPU: Self-Administered Questionnaire for Adolescent Problematic Mobile Phone Use ^83^

Table S3. Quality assessment of included studies using the Newcastle-Ottawa Scale.

|  | **Selection** | | | | **Comparability** | **Exposure** | |  | **Subtotal Assessment** | |  |
| --- | --- | --- | --- | --- | --- | --- | --- | --- | --- | --- | --- |
| Source | **Representativeness of sample** | **Ascertainment of exposure** | **Sample size** | **Non-respondents** | **Confounders are controlled for** | **Assessment of outcome** | **Statistical test** | **S Total** |  | **E Total** | **Overall** |
| **Bhatt et al., 2017** ^64^ | * |  |  |  |  |  | * | Moderate |  | Moderate | Poor |
| **Bian & Leung, 2015** ^28^ |  | * |  |  |  |  | * | Poor |  | Moderate | Poor |
| **Cerutti et al., 2016** ^73^ |  | * |  |  |  |  | * | Poor |  | Moderate | Poor |
| **Chen et al., 2017**^29^ | * | * |  |  | * |  | * | Moderate | Good | Moderate | Moderate |
| **Chen et al., 2016** ^53^ | * | * |  |  |  |  | * | Moderate |  | Moderate | Moderate |
| **De Sola, 2017** ^30^ | * | * |  |  |  |  | * | Moderate |  | Moderate | Moderate |
| **Demirci et al., 2015** ^48^ | * | * |  |  |  |  | * | Moderate |  | Moderate | Moderate |
| **Domple et al., 2017** ^31^ |  | * |  |  |  |  | * | Poor |  | Moderate | Poor |
| **Evyazlou et al., 2016** ^61^ |  | * |  |  |  |  | * | Poor |  | Moderate | Poor |
| **Garcia-Oliva et al., 2017** ^41^ |  | * |  |  |  |  | * | Poor |  | Moderate | Poor |
| **Haug et al., 2015** ^32^ |  | * |  |  |  |  | * | Poor |  | Moderate | Poor |
| **Hawi et al., 2016** ^42^ | * | * |  |  |  |  | * | Moderate |  | Moderate | Moderate |
| **Hussain et al., 2017** ^33^ |  | * |  |  |  |  | * | Poor |  | Moderate | Poor |
| **Jiang & Shi, 2016** ^57^ | * | * |  |  | * |  | * | Moderate | Good | Moderate | Moderate |
| **Jo, Na, & Kim, 2017** ^34^ |  | * |  |  |  |  | * | Poor |  | Moderate | Poor |
| **Kim et al., 2017** ^62^ | * | * |  |  | * |  | * | Moderate | Good | Moderate | Moderate |
| **Kwon & Paek, 2016** ^35^ |  | * |  |  |  |  | * | Poor |  | Moderate | Poor |
| **Lee & Lee, 2017** ^36^ | * | * |  |  |  |  | * | Moderate |  | Moderate | Moderate |
| **Lee E. B., 2015** ^37^ |  | * |  |  |  |  | * | Poor |  | Moderate | Poor |
| **Lee H et al., 2017** ^54^ |  | * |  |  |  |  | * | Poor |  | Moderate | Poor |
| **Leung, 2017** ^44^ |  | * |  |  |  |  | * | Poor |  | Moderate | Poor |
| **Li & Lu, 2017** ^65^ | * | * |  |  |  |  | * | Moderate |  | Moderate | Moderate |
| **Long et al., 2016** ^52^ | * | * |  |  | * |  | * | Moderate | Good | Moderate | Moderate |
| **Lopez-Fernandez et al., 2017** ^45^ |  | * |  |  |  |  | * | Poor |  | Moderate | Poor |
| **Miguel, 2016** ^40^ |  | * |  |  |  |  | * | Poor |  | Moderate | Poor |
| **Mohammadbeigi et al., 2016** ^49^ | * | * |  |  | * |  | * | Moderate | Good | Moderate | Moderate |
| **Nikhita et al., 2015** ^50^ | * | * | * |  |  |  | * | Good |  | Moderate | Moderate |
| **Prasad et al., 2017** ^38^ |  | * |  |  |  |  | * | Poor |  | Moderate | Poor |
| **Rajanna et al., 2016** ^66^ | * | * |  |  |  |  | * | Moderate |  | Moderate | Moderate |
| **Tahtsidou et al., 2016** ^58^ |  | * |  |  |  |  | * | Poor |  | Moderate | Poor |
| **Tao et al., 2017** ^39^ | * | * |  |  | * |  | * | Moderate | Good | Moderate | Moderate |
| **Tao et al., 2017 (b)** ^59^ | * | * |  |  | * |  | * | Moderate | Good | Moderate | Moderate |
| **Tavakolizadeh et al., 2014** ^60^ | * | * |  |  |  |  | * | Moderate |  | Moderate | Moderate |
| **Venkatesh et al., 2017** ^56^ |  | * |  |  |  |  | * | Poor |  | Moderate | Poor |
| **Wang et al., 2013** ^67^ | * | * |  |  |  |  | * | Moderate |  | Moderate | Moderate |
| **Warzecha & Pawlak, 2017** ^46^ | * | * |  |  |  |  | * | Moderate |  | Moderate | Moderate |
| **Yildirim et al., 2016** ^47^ |  | * |  |  |  |  | * | Poor |  | Moderate | Poor |
| **Yuchang et al., 2017** ^51^ |  | * |  |  |  |  | * | Poor |  | Moderate | Poor |

| * = study adequately filled criteria for this sub-domain |
| --- |
| & = 1 - 2 (poor); 3 (moderate); 4 (good) |
| ∑ = 0 (poor); 1 (moderate); 2 (good) |
| # = 0 (poor); 1 (good) |

|  | **Selection** | | | | **Comparability** | **Exposure** | | | **Subtotal Assessment** | | |  |
| --- | --- | --- | --- | --- | --- | --- | --- | --- | --- | --- | --- | --- |
| Source | **Representativeness of sample** | **Ascertainment of exposure** | **Sample size** | **Demonstration that outcome was not present at beginning of study** | **Confounders are controlled for** | **Assessment of outcome** | **Length of follow up** | **Follow up rate** | **S^&^ Total** | **C**^#^ **Total** | **E**^∑^ **Total** | **Overall** |
| **Cohort studies** | | | | | | | | | | | | |
| **Lee JE et al., 2017 ^64^** | * | * |  | * |  |  | * | * | Moderate | Poor | Good | Poor |
| **Lee H et al., 2017 (b) ^43^** |  | * |  |  |  |  |  | * | Poor | Poor | Moderate | Poor |
| **Tossell et al., 2015 ^55^** |  | * |  | * | * |  | * | * | Moderate | Good | Good | Moderate |

| * = study adequately filled criteria for this sub-domain |
| --- |
| & = 1 - 2 (poor); 3 (moderate); 4 (good) |
| ∑ = 0 (poor); 1 (moderate); 2 (good) |
| # = 0 (poor); 1 (good) |

Criteria used for Newcastle-Ottawa Quality Assessment of studies in Supplementary Table 3

If acceptable, * is given

**Cross Sectional Studies**

Selection

1. Is the sample representative?
   1. Truly representative of the average in the target population (all subjects or random sampling)*
   2. Somewhat representative of the average in the target population (non-random sampling)*
   3. Selected group of users
   4. No description of sampling strategy
2. Ascertainment of the exposure
   1. Validated measurement tool*
   2. Non-validated measurement tool, but the tool is available or described*
   3. No description of the measurement tool
3. Sample size
   1. Justified and satisfactory*
   2. Not justified
4. Non-respondents
   1. Comparability between responders and non-responders characteristics is established*
   2. The response rate is unsatisfactory or the comparability between responders and non-responders is unsatisfactory
   3. No description of the response rate or the characteristics of the responders and non-responders

Comparability

1. Controlling for confounding factors
   1. Study controls for other addictive behaviours (gaming disorder, internet addiction)*
   2. Study controls for confounding factors (age, sex, marital status, etc.) * in analysing for associated variables
   3. Study does not control for confounders

Outcome

1. Assessment of the outcome
   1. Independent blind assessment*
   2. Record linkage*
   3. Self report
   4. No description
2. Statistical test
   1. The statistical test used to analyse the data is clearly described and appropriate, and the measurement of the association is presented, including confidence intervals and the probability level*
   2. The statistical test is not appropriate, not described, or incomplete

**Cohort studies**

Selection

1. Is the sample representative?
   1. Truly representative of the average in the target population (all subjects or random sampling)*
   2. Somewhat representative of the average in the target population (non-random sampling)*
   3. Selected group of users
   4. No description of sampling strategy
2. Ascertainment of the exposure
   1. Validated measurement tool, or non-validated measurement tool, but the tool is described, available, or cited*
   2. No description of the measurement tool
3. Demonstration that outcome was not present at start of study
   1. Satisfactory*
   2. No description or not satisfactory

Comparability

1. Controlling for confounding factors
   1. Study controls for confounding factors (age, sex, marital status, etc.) *
   2. Study does not control for confounders

Outcome

1. Assessment of the outcome
   1. Independent blind assessment or record linkage*
   2. Self report or no description
2. Follow up length
   1. Sufficient for outcomes to occur*
   2. No description or insufficient
3. Follow up rate
   1. Complete follow up, or subjects lost to follow up unlikely to introduce bias (>90% or description of those lost)*
   2. No description or insufficient

Table S4. Definitions and Problematic Smartphone Usage terms used, by included studies.

| **Term** | **N** | **Studies** | **Included instrument(s)** |
| --- | --- | --- | --- |
| **Abuse** | 1 | 73 | Shorter Promis Questionnaire for mobile phone addiction ^74^ |
| **Addiction** | 15 | 28,32,35,37,43,46,48,51,54,55,56,59,64,65, | Smartphone addiction index (independent but validated); SAS; SAS-SV*; KBUTK (Mobile Phone Addiction Assessment Questionnaire)^80^; MPAS^£^; SAPS^∂^; SQAPMPU^¶^; Smartphone Addiction Measurement Index (independent) |
| **Addiction Proneness** | 2 | 34,36 | SAPS^∂^ |
| **Addiction Risk** | 1 | 42 | SAS-SV* |
| **Addictive Use** | 1 | 67 | Information Technology Addiction Survey (Indian council of medical research, 2013) |
| **Dependence** | 6 | 31,44,50,58,63,67 | Mobile Phone Dependence Scale (independent but validated); MPDQ^$^; Test of Mobile Phone Dependence, Brief ^84^ |
| **Excessive Use** | 1 | 60 | Mobile Phone Addiction Index ^79^ |
| **Nomophobia** | 2 | 38,47 | Nomophobia Questionnaire ^17^ |
| **Overuse** | 3 | 49,61,62 | Cell-phone Over-use Scale ^75^; SAPS^∂^ |
| **Problematic Use** | 9 | 30,33,39–41,45,52,57,60 | MPPUS^&^; Cuestionario de Experiencias Relacionadas con el Móvil/CERM ^76^; Problematic Smartphone Use Scale (independent); SQAPMPU^¶^; Problematic Cell Phone Use Questionnaire ^85^ |

* SAS: Smartphone Addiction Scale; SAS-SV: Smartphone Addiction Scale – Short Version ^14,18^

£ MPAS (Mobile Phone Addiction Scale) or PMPUS (Problematic Mobile Phone Use Scale), due to difference in translation from Chinese^81^

& MPPUS: Mobile Phone Problematic Use Scale ^13^

∂ SAPS: Smartphone Addiction Proneness Scale ^15^

$ MPDQ: Mobile Phone Dependence Questionnaire ^82^

¶ SQAPMPU: Self-Administered Questionnaire for Adolescent Problematic Mobile Phone Use ^83^

Table S5. Mapping the instruments used to assess Problematic Smartphone Usage assessing PSU map onto criteria for behavioural addiction

| **Studies which used this tool** | **Tool** | **Intense desire to use** | **Loss of control** | **Tolerance** | **Withdrawal** | **Neglect of other activities** | **Continued use, despite harm** |
| --- | --- | --- | --- | --- | --- | --- | --- |
| 44,82 | MPDQ | x |  |  | x | x |  |
| 13,30,40 | **MPPUS** | **x** | **x** | **x** | **x** | **x** | **x** |
| 49,61,75 | COS | x | x | x | x |  | x** |
| 60,98 | **MPAI*** | **x** | **x** | **x** | **x** | **x** | **x**** |
| 52,85 | **PCPU** | **x** | **x** | **x** | **x** | **x** | **x** |
| 31,84 | TMD | x | x | x | x |  | x |
| 18,48 | **SAS** | **x** | **x** | **x** | **x** | **x** | **x** |
| 14,29,32,37,42,43,51 | **SAS-SV*** | **x** | **x** | **x** | **x** | **x** | **x** |
| 28 | **SPAI** | **x** | **x** | **x** | **x** | **x** | **x**** |
| 50 | MPD | x | x | x | x | x |  |
| 17,47 | NMP-Q |  |  |  | x |  |  |
| 15,34,54,62,64 | SAPS | x | x |  | x | x | x** |
| 39,65,83 | SQAPMPU |  |  | x | x | x |  |
| 38 | Unnamed questionnaire tailored to medical students |  |  |  | x | x | x** |

*short or adapted form of another instrument;

** harm equated to social consequences but physical or mental health harms not incorporated into scale

**Legend:**

This table shows those questionnaires that could be assessed according to whether they considered all diagnostic criteria for a behavioural addiction, when this could be assessed. Nineteen studies used instruments which were well described and which covered all criteria for the diagnosis of a behavioural addiction (in bold). A further eleven described tools in detail which measured certain aspects of addiction, such as withdrawal phenomena, but not others, while ten studies named questionnaires but did not describe them in sufficient detail for the reader to gauge whether they mapped onto diagnostic criteria for addiction.

Table S6. Summary of the results of the included studies.

| Studies | Depression | | Stress | | | Anxiety | | Poor sleep quality | | Poor educational attainment | |
| --- | --- | --- | --- | --- | --- | --- | --- | --- | --- | --- | --- |
| Bhatt et al., 2017 ^63^ |  |  |  |  |  | |  | + | r |  |  |
| Chen et al., 2017 ^59^ | + | ¶ |  |  | + | |  | + | ¶ |  |  |
| Chen et al., 2016 ^53^ | + | **ß** |  |  |  | |  |  |  |  |  |
| Demirci et al., 2015 ^48^ | + | **r, ß** |  |  | + | | **r, ß** | o | **χ2, r, ß** |  |  |
| Eyvazlou et al., 2016 ^61^ | + | **r, ß** |  |  | + | | **r, ß** | + | **r** |  |  |
| Haug et al., 2015 ^32^ |  |  | + | ¶ |  | |  |  |  |  |  |
| Hawi et al., 2016 ^42^ |  |  |  |  |  | |  |  |  | + | ¶ |
| Hussain et al., 2017 ^33^ |  |  |  |  | + | | r |  |  |  |  |
| Kim et al., 2017 ^62^ |  |  | + | ¶ |  | |  |  |  |  |  |
| Kwon & Paek, 2016 ^35^ | + | r |  |  |  | |  |  |  | + | **ß** |
| Lee & Lee, 2017 ^36^ |  |  |  |  |  | |  |  |  | + | χ^2^ |
| Lee JE et al., 2017 ^64^ |  |  |  |  |  | |  | + | ¶ |  |  |
| Li & Lu, 2017 ^65^ |  |  |  |  |  | |  | + | r |  |  |
| Long et al., 2016 ^52^ | + | ¶ | + | ¶ | + | | ¶ |  |  |  |  |
| Mohammadbeigi et al., 2016 ^49^ |  |  |  |  |  | |  | + | ¶ |  |  |
| Tahtsidou et al., 2016 ^58^ |  |  | o | χ^2^ |  | |  |  |  |  |  |
| Tao et al., 2017 (b) ^59^ | + | ¶ |  |  | + | | ¶ |  |  |  |  |
| Tavakolizadeh et al., 2014 ^60^ | + | χ^2^ |  |  | - | | χ^2^ |  |  | o | χ^2^ |
| Venkatesh et al., 2017 ^56^ |  |  | + | r |  | |  |  |  |  |  |
| Wang et al., 2013 ^67^ |  |  |  |  |  | |  |  |  | + | ¶ |

| Key | | | |
| --- | --- | --- | --- |
|  | Type of association reported |  | Measure of effect used |
| + | Significant positive association | ¶ | Odds ratio |
| - | Significant negative association | **ß** | Regression coefficient |
| o | No significant association | **r** | Correlation coefficient |
|  |  | χ^2^ | Chi-squared value |

Additional References (Supplementary Tables 4-5):

73. Cerutti R, Presaghi F, Spensieri V, Valastro C, Guidetti V. The Potential Impact of Internet and Mobile Use on Headache and Other Somatic Symptoms in Adolescence. A Population-Based Cross-Sectional Study. *Headache*. 2016;56(7):1161-1170. doi:10.1111/head.12840

74. Mimma T, Baiocco R. Addictive Behavior and Family Functioning During Adolescence. *Am J Fam Ther*. 2009;37(5):388-395. doi:10.1080/01926180902754745

75. Jenaro C, Flores N, Gómez-Vela M, González-Gil F, Caballo C. Problematic internet and cell-phone use: Psychological, behavioral, and health correlates. *Addict Res Theory*. 2007;15(3):309-320. doi:10.1080/16066350701350247

76. Beranuy Fargues M, Chamarro Lusar A, Graner Jordania C, Carbonell Sanchez X. Validation of two brief scales for Internet addiction and mobile phone problem use. *Psicothema*. 2009;21(3):480-485.

77. Billieux J, Van der Linden M, Rochat L. The role of impulsivity in actual and problematic use of the mobile phone. *Appl Cogn Psychol*. 2008;22(9):1195-1210. doi:10.1002/acp.1429

78. Aggarwal M, Grover S, Basu D. Mobile phone use by resident doctors: Tendency to addiction-like behaviour. *Ger J Psychiatry*. 2012;15(2):50-55.

79. Leung L. Linking psychological attributes to addiction and improper use of the mobile phone among adolescents in Hong Kong. *J Child media*. 2008;2(2):93-113.

80. Pawlowska B, Potembska E. Właściwości psychometryczne Kwestionariusza do Badania Uzależnienia od Telefonu Komórkowego (KBUTK). *Badania nad Schizofr*. 2009;10:322-329.

81. Xiong J, Zhou Z, Chen W, You Z, Zhai Z. Development of the mobile phone addiction tendency scale for college students. *Chinese Ment Heal J*. 2012;26:222-225.

82. Toda M, Monden K, Kubo K, Morimoto K. Cellular phone dependence tendency of female university students. *Nihon eiseigaku zasshiJapanese J Hyg*. 2004;59(4):383-386.

83. Tao S, Fu J, Wang H, Hao J, Tao F. The development of Self-rating Questionnaire for Adolescent Problematic Mobile Phone Use and the psychometric evaluation in undergraduates. *Chin J Sch Heal*. 2013;34(1):26-29.

84. Choliz M, Pinto L, Phansalkar SS, et al. Development of a Brief Multicultural Version of the Test of Mobile Phone Dependence (TMDbrief) Questionnaire. *Front Psychol*. 2016;7:650. doi:10.3389/fpsyg.2016.00650

85. Yen CF, Tang TC, Yen JY, et al. Symptoms of problematic cellular phone use, functional impairment and its association with depression among adolescents in Southern Taiwan. *J Adolesc*. 2009;32(4):863-873. doi:10.1016/j.adolescence.2008.10.006
