# Supplementary material for: Prevalence of problematic smartphone usage and associated mental health outcomes amongst children and young people: a systematic review, meta-analysis and GRADE of the evidence
Source: BMC Psychiatry. 2019 Nov 29;19:356. doi: 10.1186/s12888-019-2350-x (PMC6883663; doi:10.1186/s12888-019-2350-x)

Figure S1. Meta-analyses of Problematic Smartphone Usage (PSU) and the secondary educational outcomes.


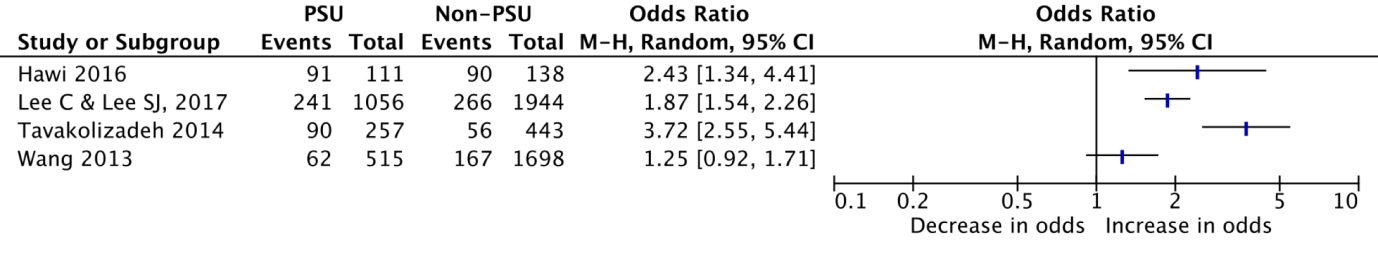

Supplement: Supplementary file 2 — Additional file 2 Figure S1. Meta-analyses of Problematic Smartphone Usage (PSU) and the secondary educational outcomes. [file 12888_2019_2350_MOESM2_ESM.docx]
